# Papéis na organização, conflito trabalho-família, satisfação laboral e saúde mental de docentes em relação com o comprometimento organizacional afetivo

**DOI:** 10.1590/0102-311XPT143723

**Published:** 2024-11-04

**Authors:** Virgínia D. Carvalho

**Affiliations:** 1 Universidade Federal de Alfenas, Varginha, Brasil.

**Keywords:** Papel Profissional, Conflito de Papéis, Satisfação no Emprego, Saúde Mental, Engajamento no Trabalho, Professional Role, Role Conflict, Job Satisfaction, Mental Health, Work Engagement, Rol Profesional, Conflicto de Roles, Satisfacción en el Trabajo, Salud Mental, Compromiso Laboral

## Abstract

Com o objetivo de examinar relações múltiplas entre papéis na organização, conflito trabalho-família, satisfação laboral, saúde mental e comprometimento organizacional afetivo entre docentes da educação básica, realizou-se um estudo transversal com amostra de 438 respondentes, aos quais foram aplicadas a *Escala para Avaliação de Estressores Psicossociais no Contexto Laboral* (EAEPCL), a *Escala de Satisfação Geral no Trabalho*, o *Questionário de Saúde Geral* (QSG-12) e a *Escala Reduzida de Comprometimento Organizacional Afetivo* (ECOA). Foi empregada a modelagem de equações estruturais para a análise dos dados, e a estimação do modelo de mensuração indicou validade convergente e discriminante e confiabilidade no que tange às variáveis latentes utilizadas. Efeitos diretos do conflito e da ambiguidade de papéis em relação à satisfação laboral, aos transtornos mentais comuns (TMC) e ao comprometimento organizacional afetivo foram observados, bem como efeitos indiretos sobre essas duas últimas com a mediação da satisfação laboral. Quanto à sobrecarga de papéis, seus efeitos diretos aconteceram apenas em relação à satisfação laboral, sendo que essa última mediou o efeito da primeira sobre os TMC e o comprometimento afetivo, ao passo que o conflito trabalho-família mediou o efeito da sobrecarga de papéis na ocorrência de TMC. Os resultados contribuem para salientar os efeitos nocivos dos estressores de papel e da (in)satisfação laboral sobre a saúde mental dos pesquisados ao torná-los mais vulneráveis ao desenvolvimento de TMC e sinalizam como eles podem afetar os resultados de interesse das instituições estudadas, como o comprometimento afetivo em relação às organizações escolares.

## Abstract (en)

To examine multiple relations between organizational roles, work-family conflict, job satisfaction, mental health, and affective organizational commitment among basic education teachers, a cross-sectional study was conducted with a sample of 438 respondents, to whom the *Scale for the Assessment of Psychosocial Stressors in the Workplace* (EAEPCL, acronym in Portuguese), the *General Job Satisfaction Scale*, the *General Health Questionnaire* (GHQ-12) and the *Reduced Affective Organizational Commitment Scale* (ECOA, acronym in Portuguese) were applied. Data were analyzed by structural equation modeling and the estimated measurement model indicated convergent and discriminant validity, as well as reliability regarding the latent variables used. Direct effects of conflict and role ambiguity on job satisfaction, common mental disorders (CMD) and affective organizational commitment were observed, as well as indirect effects on the latter two mediated by job satisfaction. As for role overload, its direct effects occurred only in relation to job satisfaction, the latter mediating the effect of the former on CMD and affective commitment, whereas work-family conflict mediated the effect of role overload on CMD. These results highlight the harmful effects of role stressors and job (dis)satisfaction on the mental health of teachers by making them more vulnerable to developing CMD and indicate how these can affect results of interest to the institutions studied, such as affective commitment in school organizations.

## Resumen

Con el objetivo de examinar múltiples relaciones entre papeles en la organización, conflicto trabajo-familia, satisfacción laboral, salud mental y compromiso organizacional afectivo entre docentes de educación básica, se realizó un estudio transversal con una muestra de 438 encuestados, a quienes se les aplicó la *Escala de Evaluación de Estresores Psicosociales en el Contexto Laboral* (EAEPCL, por su sigla en portugués), la *Escala de Satisfacción General en el Trabajo*, el *Cuestionario de Salud General* (QSG-12, por sus siglas en portugués) y la *Escala Reducida de Compromiso Organizacional Afectivo* (ECOA, por sus siglas en portugués). Para el análisis de los datos se empleó el modelado de ecuaciones estructurales, y la estimación del modelo de medición indicó validez convergente y discriminante, así como confiabilidad con respecto a las variables latentes utilizadas. Se observaron efectos directos del conflicto y la ambigüedad de papel con relación a la satisfacción laboral, los trastornos mentales comunes (TMC) y el compromiso afectivo organizacional, así como efectos indirectos sobre estos dos últimos con la mediación de la satisfacción laboral. En cuanto a la sobrecarga de papeles, sus efectos directos se dieron solo con relación a la satisfacción laboral, y esta última es la que medió el efecto de la primera sobre los TMC y el compromiso afectivo, mientras que el conflicto trabajo-familia medió el efecto de la sobrecarga de papeles en la incidencia de TMC. Los resultados contribuyen para resaltar los efectos nocivos de los estresores de papel y de la (in)satisfacción laboral en la salud mental de los encuestados al hacerlos más vulnerables al desarrollo de TMC y señalan cómo estos pueden afectar resultados de interés para las instituciones estudiadas, como el compromiso afectivo con relación a las organizaciones escolares.

## Introdução

A intensificação do trabalho docente, especialmente quando se considera o contexto da educação básica, é uma realidade que vem sendo discutida por vários estudiosos. Destacam-se, nesse debate, a expansão do espaço de atuação da escola, que amplia os papéis docentes, além do maior volume de atividades administrativas, resultando em um cenário de aumento das exigências sobre os professores, que passam a ser crescentemente responsabilizados pelos resultados escolares, sem que haja contrapartida em termos de investimentos que proporcionem adequadas condições de trabalho ^1^ ^,^ ^2^ ^,^ ^3^ ^,^ ^4^ ^,^ ^5^. Nesse cenário, variáveis como sobrecarga, conflito e ambiguidade de papéis tendem a emergir como estressores que configuram risco psicossocial, podendo causar desdobramentos para o bem-estar desses trabalhadores.

Isso ocorre porque o conflito e a ambiguidade de papéis, ao representarem, respectivamente, as pressões ocasionadas pela exigência do desempenho de papéis incompatíveis e falta de clareza quanto ao que é esperado do professor, traduzem-se em fontes de tensão no trabalho. No que tange, por sua vez, à sobrecarga de papéis, configura um problema que chega a extrapolar o ambiente laboral, interferindo em outras esferas da vida dos professores, pois, conforme já pontuaram Assunção & Abreu ^2^, estabelece uma concorrência de tempo entre atividades da escola e espaço da família, o que pode ocasionar um desequilíbrio, dada a restrição dos períodos de descanso e recuperação, com riscos evidentes à saúde dos docentes e prejuízos à qualidade do ensino. É nessa vertente que Araújo et al. ^1^ já defendiam a ampliação do foco da análise quando se estuda a saúde docente, incorporando discussões como a do conflito trabalho-família e suas consequências para essa categoria profissional, o que pressupõe considerar não somente o problema da escassez de tempo, mas a possibilidade de que as tensões provenientes do trabalho contaminem a qualidade das relações no lar.

O sofrimento decorrente desse processo de desgaste laboral tem sido apontado em estudos como o de Luz et al. ^6^, que identificaram, por meio de revisão sistemática da literatura, as formas mais frequentes de adoecimento entre os professores, com destaque para os transtornos mentais e psicossomáticos, devidos, principalmente, às más condições de trabalho no ambiente escolar. Entre esses, destaca-se a elevada prevalência de transtornos mentais comuns (TMC), atestada por numerosas pesquisas ^5^ ^,^ ^6^ ^,^ ^7^ ^,^ ^8^ ^,^ ^9^. Há, ainda, estudos como os de Ferreira-Costa & Pedro-Silva ^10^ e Viana et al. ^11^, que problematizaram os impactos do referido contexto laboral sobre a (in)satisfação no trabalho docente.

Entretanto, embora haja indicações da literatura de que os estressores de papel e o conflito trabalho-família tendem a se associar não somente à ocorrência de transtornos mentais, mas também à diminuição da satisfação no trabalho ^12^ ^,^ ^13^ ^,^ ^14^ ^,^ ^15^ ^,^ ^16^, afetando, ainda, o comprometimento afetivo com a organização ^16^ ^,^ ^17^ ^,^ ^18^ ^,^ ^19^ ^,^ ^20^. A relação entre tais variáveis demanda maior exploração junto ao público docente. Isso implica uma necessidade de se problematizar não apenas o impacto dos referidos estressores sobre as variáveis mencionadas, mas conhecer melhor a relação que se estabelece entre a satisfação no trabalho, a ocorrência de TMC e o comprometimento afetivo com a organização, no contexto de professores atuantes na educação básica.

Tal demanda se sustenta pelo fato de que a satisfação no trabalho, sendo um dos domínios relevantes do bem-estar subjetivo ^21^, pode favorecer melhorias nos indicadores de saúde física e mental dos trabalhadores ^22^ ^,^ ^23^. É também conhecida a sua relação com o comprometimento afetivo com a organização ^24^ ^,^ ^25^ ^,^ ^26^, o qual, sendo uma espécie de vínculo estabelecido com base na identificação e envolvimento do indivíduo com a organização ^27^, costuma se manifestar na forma de empenho e interesse na execução das atividades ^24^ ^,^ ^28^. No contexto de atuação docente, isso se traduz em termos de impactos positivos na qualidade da educação ^29^, uma vez que o comprometimento dos professores tende a se relacionar positivamente ao aprendizado dos alunos ^30^ ^,^ ^31^, além de contribuir para outros resultados relevantes, como maiores níveis de engajamento no trabalho e intenção de permanência ^32^. E, embora haja ainda poucas incursões que contemplaram a relação entre o comprometimento organizacional afetivo e a saúde mental no trabalho docente ^32^ ^,^ ^33^, elas são indicadoras da pertinência de se estudar tal associação.

Conforme indicaram Cooper et al. ^34^, ao analisar as relações entre fatores de risco psicossocial no trabalho e as respostas mais comumente associadas a eles, é importante que sejam também examinadas as relações entre tais respostas. Segundo os autores, isso pode contribuir para compreender se a experiência de um tipo de reação psicológica pode tornar os indivíduos mais vulneráveis a outros tipos de respostas dessa natureza, enriquecendo a compreensão do fenômeno.

Reunindo, portanto, algumas variáveis que emergem a partir dessa contextualização inicial, as quais assumem centralidade por tratarem de elementos que caracterizam a rotina de trabalho docente, e considerando a importância de se evidenciar, não somente o nexo causal entre os riscos psicossociais aqui focalizados e o adoecimento psíquico, como também o seu impacto sobre os indicadores de bem-estar e as possíveis relações desses últimos com a saúde mental, é que se destaca a pertinência de compreender a dinâmica que se estabelece entre elas. Com esse intuito, desenhou-se o objetivo deste estudo, que se propôs a avaliar as relações múltiplas entre os papéis na organização, o conflito trabalho-família, a satisfação laboral, a saúde mental e o comprometimento organizacional afetivo entre docentes atuantes na educação básica pública, ofertada pela rede estadual de ensino na região sul de Minas Gerais, Brasil.

## Método

O estudo exploratório, de corte transversal, é parte de um projeto maior denominado *Docentes e Saúde Psíquica no Trabalho* que avaliou a saúde psíquica laboral entre professores da educação básica pública a partir do exame de diferentes variáveis contextuais e individuais e teve sua versão preliminar apresentada em congresso ^35^. A população do estudo envolveu a totalidade dos docentes (792 sujeitos) atuantes nas 19 escolas da rede estadual de dois municípios da região sul de Minas Gerais, os quais não foram identificados por exigência da Superintendência Regional de Ensino, que autorizou a pesquisa. Dessa população, originou-se uma amostra não probabilística por conveniência de 438 respondentes, que se dispuseram voluntariamente a participar da pesquisa, tendo como único critério de inclusão o tempo mínimo de um ano de serviço como docente.

Com vistas a atender ao objetivo deste estudo, foram utilizadas as informações obtidas a partir da aplicação de cinco instrumentos. As variáveis que abordaram os papéis na organização e o conflito trabalho-família foram levantadas por meio da *Escala para Avaliação de Estressores Psicossociais no Contexto Laboral* (EAEPCL) ^36^; os indicadores de TMC e de satisfação laboral foram acessados, respectivamente, pelo *Questionário de Saúde Geral* (QSG-12) de Goldberg ^37^ e pela *Escala de Satisfação Geral no Trabalho* ^38^; os níveis de comprometimento foram avaliados com o uso da *Escala de Comprometimento Organizacional Afetivo* (ECOA) em sua versão reduzida ^39^ ^,^ ^40^; e um formulário sociodemográfico e laboral permitiu conhecer aspectos concernentes ao perfil dos docentes, indagando sobre idade, sexo, estado civil, renda, escolaridade, tipo de vínculo com a organização, tempo de serviço, número de escolas em que atuava e número médio de alunos por turma.

A EAEPCL foi construída e testada por Ferreira et al. ^36^, fundamentando-se no modelo de Cooper et al. ^34^, e apresenta 35 itens, cujas respostas são assinaladas em escala de 1 (nunca) até 6 (sempre), que indica a intensidade com que o indivíduo percebe cada estressor apontado como algo presente no seu contexto de trabalho e que lhe transmite algum tipo de mal-estar. Esses itens são distribuídos em sete fatores, que consistem nas dimensões de estressores mensuradas pelo referido instrumento, quais sejam: conflito e ambiguidade de papéis; sobrecarga de papéis; falta de suporte social; insegurança na carreira; falta de autonomia; conflito trabalho-família; e pressão do grau de responsabilidade. As evidências apontadas no estudo original de validação da escala foram indicativas de aceitável nível de consistência dos fatores mencionados, com alfas de Cronbach entre 0,72 e 0,82. A EAEPCL foi aplicada integralmente junto aos respondentes; nesta análise, porém, foram empregados apenas os itens referentes às dimensões conflito e ambiguidade de papéis, sobrecarga de papéis e conflito trabalho-família, que consistiam nas variáveis latentes analisadas neste estudo.

Quanto à utilização do QSG-12, que costuma ser apresentado como uma medida de bem-estar psicológico, visou ao levantamento dos indicadores de TMC, por meio de uma versão adaptada e testada no Brasil por Borges & Argolo ^37^. O referido instrumento de diagnóstico avalia sintomas e comportamentos que sejam indicativos de transtornos psíquicos leves. Os itens são assinalados em uma escala de 0 a 3 pontos que se referem à intensidade com que cada um dos sintomas apontados é experimentado pelo respondente. Dado que não há consenso na literatura quanto à sua estrutura fatorial, sendo admitidas soluções unifatoriais ou bifatoriais ^37^ ^,^ ^41^, optou-se por operacionalizá-lo neste estudo de maneira unifatorial, estrutura a qual já havia apresentado confiabilidade elevada anteriormente, haja vista o coeficiente alfa de Cronbach da ordem de 0,88 obtido quando da extração de um único fator no estudo de validação empreendido por Borges & Argolo ^37^.

A *Escala de Satisfação Geral no Trabalho*, por sua vez, foi desenvolvida por Silva & Ferreira ^38^ e se trata de instrumento unidimensional, composto por cinco itens que são respondidos em uma escala variando de 1 a 6 pontos (desde “discordo fortemente” até “concordo fortemente”), expressando o nível de satisfação com o trabalho por parte do pesquisado. As autoras do instrumento obtiveram índice de consistência interna satisfatório para essa escala, tendo apresentado originalmente coeficiente alfa de Cronbach de 0,80 ^38^, mostrando-se, assim, uma medida parcimoniosa de satisfação no trabalho que tem sido empregada em outros estudos no tema ^36^ ^,^ ^42^ ^,^ ^43^.

Igualmente, a Ecoa em sua versão reduzida de cinco itens, construída e testada por Siqueira ^39^ e apresentada por Bastos et al. ^40^, é uma medida unidimensional bem aceita na literatura ^26^ ^,^ ^44^ ^,^ ^45^, possuindo itens estruturados no sentido de indicar em uma escala de 1 a 5 pontos (variando de “nada” a “extremamente”) os sentimentos positivos e negativos que representam o vínculo afetivo do respondente com relação à organização em que trabalha. O instrumento se caracteriza por alto índice de precisão (alfa de Cronbach = 0,93), além de apresentar correlação elevada (r = 0,95; p < 0,01) com a sua versão completa ^40^, o que o torna uma alternativa concisa para a mensuração do nível de comprometimento organizacional afetivo dos trabalhadores.

A coleta de informações foi desenvolvida no âmbito do projeto supramencionado e envolveu contato com a Superintendência Regional de Ensino e as escolas participantes para obter autorização e realizar o agendamento da apresentação da pesquisa e da aplicação dos questionários junto aos docentes. Foi assegurada a preservação da identidade dos participantes ao longo de todo o levantamento, a análise e a divulgação dos resultados, sendo que a eles foi apresentado, em duas vias de igual teor, um Termo de Consentimento Livre e Esclarecido (TCLE), o qual leram e assinaram, ficando uma das vias em posse do respondente e a outra do coordenador da pesquisa. A operacionalização do levantamento aconteceu por meio da distribuição de questionários impressos que foram respondidos durante os minutos iniciais de reuniões pedagógicas nas instituições envolvidas e ocorreu, principalmente, entre os meses de fevereiro e março de 2019. Ao final do processo, foi obtido um total de 452 questionários preenchidos, tendo sido descartados 14 por preenchimento incompleto, contando-se um total de 438 questionários válidos.

O tratamento das informações teve início com o emprego de estatísticas descritivas (análises de frequência, média e desvio padrão) para o desenho do perfil sociodemográfico e laboral dos respondentes. Na sequência, foi utilizada a modelagem de equações estruturais (MEE), que consiste em “uma técnica de modelagem estatística multivariada de caráter geral”, frequentemente apontada como uma combinação de aspectos de análise fatorial e regressão múltipla ^46^, para a análise das relações de dependência entre as variáveis do estudo. A MEE foi realizada por meio do software SmartPLS 3.3.2 (https://www.smartpls.com/) e compreendeu as etapas de avaliação do modelo de mensuração e estimação do modelo estrutural, por mínimos quadrados parciais.

Este trabalho foi desenvolvido em conformidade com os requisitos da *Resolução nº 466*, de 12 de dezembro de 2012, do Conselho Nacional de Saúde, tendo sido aprovado pelo Comitê de Ética em Pesquisa da Universidade Federal de Alfenas (parecer n^o^ 3.156.204).

## Resultados

Partindo de uma população de 792 docentes, que constituíam o quantitativo de professores atuantes nas 19 escolas envolvidas no estudo, chegou-se a uma amostra não probabilística por conveniência de 452 respondentes, composta por aqueles que se voluntariaram a participar da pesquisa. Entre eles, entretanto, 14 preencheram os questionários de forma incompleta, inviabilizando sua inclusão nas análises, de modo que a amostra final ficou reduzida a 438 respondentes. Essa amostra foi composta predominantemente por mulheres (73,7%), com faixa etária entre 31 e 50 anos (67,7%), a maioria casada (58,1%) e com rendimentos mensais variando de 1 a 3 salários mínimos (49,8%) e 3 a 5 salários mínimos (35,9%). Quanto à escolaridade, destacaram-se as formações em nível de graduação (34,9%) e especialização (55,3%). O vínculo empregatício mais comum foi o efetivo (63,2%) e o tempo total de serviço oscilou entre 1 e 44 anos (M = 14,51 anos/desvio padrão - DP = 8,45). Aproximadamente a metade desses docentes (46,3%) relatou trabalhar em duas escolas e o número médio de alunos por turma esteve na faixa de 34,13 (DP = 6.74).

Os resultados concernentes à estimação do modelo de mensuração foram indicativos de validade convergente e discriminante e também de confiabilidade no que se refere às variáveis latentes (VL) utilizadas no modelo. Conforme pode ser observado na Tabela 1, a variância média extraída (*average variance extracted* - AVE) apresentou valores superiores a 0,5 para todas as VL, a raiz quadrada da AVE foi maior do que os coeficientes de correlação e os indicadores de confiabilidade composta maiores do que 0,85, além dos coeficientes alfa entre 0,78 e 0,94.

**Tabela 1:** Resultados do modelo de mensuração.

	1	2	3	4	5	6
1. Conflito e ambiguidade de papéis	0,785					
2. Conflito trabalho-família	0,324	0,728				
3. Transtornos mentais comuns	0,343	0,508	0,742			
4. Comprometimento afetivo	-0,370	-0,278	-0,463	0,895		
5. Sobrecarga de papéis	0,106	0,578	0,362	-0,217	0,726	
6. Satisfação no trabalho	-0,257	-0,350	-0,546	0,625	-0,283	0,822
Alfa de Cronbach	0,843	0,781	0,925	0,937	0,830	0,880
Confiabilidade composta	0,889	0,849	0,936	0,952	0,867	0,913
Variância média extraída	0,616	0,529	0,550	0,800	0,527	0,676

**Figura 1:**
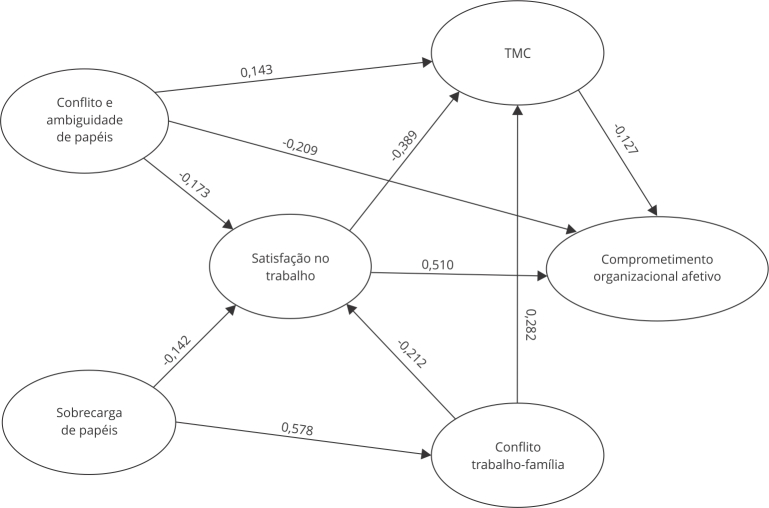
Modelo estrutural.

Avaliando o modelo estrutural (Tabelas 2 e 3, Figura 1), os resultados apontaram os efeitos diretos, indiretos e totais das variáveis preditoras em relação às endógenas. Os efeitos diretos são apresentados na Tabela 2, na qual se observa a associação negativa entre as variáveis de papel (conflito e ambiguidade de papéis e sobrecarga de papéis) e a satisfação no trabalho. A capacidade explicativa foi da ordem de 15,2% e os coeficientes estruturais indicaram que a variável de papel com associação negativa à satisfação no trabalho de maior magnitude foi o conflito e a ambiguidade de papéis. No que se refere à capacidade explicativa oferecida aos TMC e ao comprometimento afetivo, ocorreu apenas a partir da variável conflito e ambiguidade de papéis, com uma variância explicada bastante expressiva (42,5% e 44% respectivamente), ainda que o tamanho do efeito (f^2^ = 0,03 e f^2^ = 0,06, respectivamente) tenha sido pequeno.

**Tabela 2:** Resultados do modelo estrutural - efeitos diretos.

	Coeficiente estrutural	Valor-t	Valor de p	f^2^	R^2^ ajustado
CAP → ST	-0,173	3,047	0,002	0,032	0,152
SP → ST	-0,142	2,301	0,021	0,016	
CAP → TMC	0,143	3,268	0,001	0,031	0,425
SP → TMC	0,074	1,523	0,128	0,006	
CAP → COAf	-0,209	4,218	0,000	0,066	0,440
SP → COAf	-0,035	0,713	0,476	0,001	

CAP: conflito e ambiguidade de papéis; SP: sobrecarga de papéis; ST: satisfação no trabalho; TMC: transtornos mentais comuns; COAf: comprometimento organizacional afetivo.

Nota: os valores de *variance inflation factor* (VIF) apresentaram valor mínimo de 1 e máximo de 1,87, não havendo, portanto, indicativo de multicolinearidade.

**Tabela 3:** Resultados do modelo estrutural - efeitos indiretos e totais.

Efeitos	Coeficiente	Erro padrão	Valor-t	Valor de p
SP → CTF → ST	-0,123	0,040	3,063	0,002
Total: SP → ST	-0,265	0,048	5,469	0,000
SP → CTF → TMC	0,163	0,032	5,084	0,000
SP → CTF → ST → TMC	0,048	0,018	2,719	0,007
SP → ST → TMC	0,055	0,025	2,200	0,028
Total SP → TMC	0,340	0,041	8,274	0,000
SP → CTF → COAf	0,031	0,033	0,921	0,357
SP → CTF → TMC → COAf	-0,021	0,010	2,127	0,033
SP → CTF → ST → COAf	-0,063	0,022	2,829	0,005
SP → CTF → ST → TMC → COAf	-0,006	0,004	1,728	0,084
SP → ST → TMC → COAf	-0,007	0,004	1,585	0,113
SP → ST → COAf	-0,072	0,032	2,257	0,024
Total SP → COAf	-0,173	0,049	3,719	0,000
CAP → ST → TMC	0,067	0,023	2,875	0,004
Total CAP → TMC	0,211	0,050	4,231	0,000
CAP → ST → TMC → COAf	-0,009	0,005	1,777	0,076
CAP → ST → COAf	-0,088	0,030	2,993	0,003
CAP → TMC → COAf	-0,018	0,010	1.892	0,058
Total CAP → COAf	-0,324	0,060	5,411	0,000

CAP: conflito e ambiguidade de papéis; CTF: conflito trabalho-família; SP: sobrecarga de papéis; ST: satisfação no trabalho; TMC: transtornos mentais comuns; COAf: comprometimento organizacional afetivo.

TMC: transtornos mentais comuns.

Portanto, conforme o esperado, os estressores de papéis, de modo geral, demonstraram contribuir para a redução da satisfação laboral entre os docentes pesquisados. Entretanto, contrariamente ao suposto, a sobrecarga não exerceu efeito direto significativo sobre os TMC ou sobre o comprometimento afetivo. Apenas a existência de maior conflito e ambiguidade de papéis se mostrou relacionada à maior ocorrência de TMC e a menores níveis de comprometimento afetivo com a organização escolar, sendo seu efeito maior em relação a esta última comparativamente às demais variáveis endógenas. O conjunto desses resultados indica a importância dessa variável para a explicação das variáveis endógenas aqui focalizadas.

No que tange aos efeitos indiretos (bem como aos totais), estão dispostos na Tabela 3. No que concerne à relação entre a sobrecarga de papéis e a satisfação no trabalho, percebeu-se uma mediação parcial pela variável conflito trabalho-família. Considerando-se, por sua vez, a relação entre a sobrecarga de papéis e os TMC e entre a primeira e o comprometimento organizacional afetivo, constata-se a ocorrência de uma mediação total. Isso ocorreu porque a sobrecarga de papéis não apresentou efeito direto estatisticamente significativo sobre as variáveis referidas (TMC e comprometimento afetivo), mas demonstrou exercer efeito indireto sobre ambas.

No caso dos TMC como variável endógena, a mediação ocorreu por meio do conflito trabalho-família e da satisfação no trabalho. Entretanto, como mostram os coeficientes, o efeito de mediação da variável conflito trabalho-família foi maior, comparativamente ao observado para a satisfação no trabalho, sinalizando o risco que representa para o desenvolvimento de problemas de saúde mental. Quanto aos efeitos indiretos na relação entre a sobrecarga de papéis e o comprometimento organizacional afetivo, nota-se que apenas algumas variáveis ofereceram mediação estatisticamente significativa, como é o caso da satisfação no trabalho e da combinação entre essa e o conflito trabalho-família, além da combinação entre esta última e os TMC. Todas essas mediações observadas, porém, apresentaram coeficientes relativamente baixos.

Portanto, os efeitos indiretos da sobrecarga de papéis, seja contribuindo para o desenvolvimento de sintomas de TMC ou para o decréscimo dos níveis de comprometimento organizacional afetivo, devem ser considerados; com destaque para o maior coeficiente estrutural total em relação aos TMC e à satisfação no trabalho, o que remete à importância desses efeitos indiretos para a promoção de mal-estar no trabalho entre os docentes pesquisados.

No que se refere à variável conflito e ambiguidade de papéis, seus efeitos sobre os TMC foram mediados pela satisfação no trabalho, a exemplo do que também ocorreu com o comprometimento, uma vez que os TMC não ofereceram mediação estatisticamente significativa nessa relação. Cabe salientar, ainda, que as mediações foram parciais e que, contrariamente ao que ocorreu com a sobrecarga de papéis, a variável conflito e ambiguidade de papéis mostrou maior coeficiente estrutural total na predição do comprometimento organizacional afetivo, comparativamente aos TMC, sinalizando seu maior risco em termos de prejuízos à vinculação afetiva dos docentes com as escolas.

O conjunto de todos esses resultados está sistematizado na Figura 1, que ilustra a dinâmica estabelecida entre as variáveis estudadas, elucidando as múltiplas relações entre os papéis na organização escolar, o conflito trabalho-família, a satisfação no trabalho, a saúde mental e o comprometimento organizacional afetivo; em sua contribuição para compreender como a (in)satisfação laboral pode tornar os docentes mais vulneráveis ao desenvolvimento de TMC e como esses últimos aliados à (in)satisfação laboral podem torná-los mais propensos a um menor comprometimento afetivo com a organização.

## Discussão

Em conformidade com o esperado, os estressores de papéis, os quais descrevem as expectativas sociais que exercem algum tipo de pressão sobre o indivíduo ^47^, demonstraram contribuir direta e/ou indiretamente para o aumento de TMC e para a redução da satisfação laboral e do comprometimento organizacional afetivo dos docentes participantes da pesquisa. Merece destaque, em termos de efeitos diretos, a importância da variável conflito e ambiguidade de papéis na explicação das variáveis endógenas aqui focalizadas. Isso sinaliza como a existência de demandas incompatíveis sobre esses trabalhadores (conflito), aliada à imprevisibilidade e à falta de informações necessárias para o desempenho das funções e responsabilidades deles esperadas (ambiguidade) ^34^ ^,^ ^48^ podem ser danosas ao bem-estar dos docentes e à sua vinculação afetiva com as escolas. Além disso, como foi possível notar no exame de seus efeitos totais (diretos e indiretos), tendeu a ser ainda mais prejudicial ao comprometimento afetivo com a organização escolar do que à saúde mental dos docentes.

No que tange a esse último aspecto, os resultados corroboraram achados de pesquisas anteriores ^17^ ^,^ ^19^ ^,^ ^20^ as quais, ainda que não tenham focalizado o público docente, retrataram os efeitos adversos do conflito e da ambiguidade de papéis sobre o comprometimento afetivo dos trabalhadores com a organização, sendo que, nos estudos de Addae et al. ^17^ e Morrissette & Kisamore ^20^, ocorreram de forma direta e, no de Antón ^18^, de forma indireta, por meio da satisfação no trabalho, a exemplo do observado neste estudo.

Quanto à sobrecarga de papéis, é um tipo de estressor da docência apontado em outros estudos, como o de Carlotto et al. ^49^, sinalizando as inúmeras atribuições conferidas a esses profissionais, entre as quais estão, como pontuam Souza & Ramos ^4^ e Tostes et al. ^5^, o estímulo à aprendizagem dos discentes, a preparação de alunos para a vida em sociedade, a promoção da articulação escola/comunidade, além da sua atualização profissional.

A despeito de tal fato, demonstrou exercer efeito direto apenas sobre a satisfação laboral, ao contrário do esperado, sendo que, com relação às demais, exerceu apenas efeitos indiretos, ainda que expressivos. Tal resultado se torna compreensível quando se considera que tal sobrecarga, ao representar um volume de demandas para as quais não se dispõe de tempo e recursos suficientes ^12^ ^,^ ^34^ ^,^ ^47^, tende não somente a gerar uma pressão de tempo sobre os trabalhadores, como também a criar uma incerteza quanto à possibilidade de se desempenhar os papéis de maneira adequada34. Isso pode contribuir para explicar o fato de que seus efeitos totais tenham sido maiores em termos de prejuízos ao bem-estar dos participantes do estudo, com danos à sua satisfação laboral e, principalmente, à saúde mental.

A esse respeito, cumpre destacar o papel significativo de mediação total desempenhado pela variável conflito trabalho-família, tanto na relação entre a sobrecarga de papéis e a satisfação laboral quanto entre a primeira e os TMC, conforme o suposto. Vale lembrar que o conflito trabalho-família é um estressor que emerge da necessidade de gerir demandas laborais e deveres/responsabilidades familiares ^34^ ^,^ ^48^, já tendo sido observada sua relação negativa com a satisfação no trabalho em estudo conduzido por Jamshed et al. ^16^ junto a profissionais da saúde. Importa, ainda, esclarecer que, quando o conflito trabalho-família se configura por razões de tempo (como foi o caso aqui analisado), implica em uma invasão do espaço da casa pelas demandas do emprego, justamente por que essas últimas se avolumam, produzindo uma sobrecarga.

De fato, isso caracteriza a rotina dos docentes, uma vez que levam, normalmente, grande quantidade de trabalho da escola para casa, privando-os da convivência familiar, mesmo em finais de semana ou feriados, como também indicado por outros autores ^4^ ^,^ ^49^. Tem-se, portanto, nos achados deste estudo que a mediação oferecida pelo conflito trabalho-família entre a sobrecarga de papéis e o mal-estar entre os pesquisados, principalmente em relação aos TMC, tendo sido constatada a partir de coeficientes expressivos, evidencia seus riscos para a saúde mental, fortalecendo achados de outras pesquisas ^14^ ^,^ ^15^.

No que se refere à mediação oferecida pela satisfação no trabalho, contribuiu para corroborar o que apresenta a literatura sobre essa variável, tanto no que se refere aos impactos de elementos do contexto de trabalho docente sobre a (in)satisfação laboral ^10^ ^,^ ^11^ quanto no que se concerne aos efeitos que a satisfação laboral tende a exercer, de modo geral, em termos de saúde física e mental dos trabalhadores em diversas áreas ^22^ ^,^ ^23^. Além disso, fortalece os achados de pesquisas como as de Lizote et al. ^25^ e Traldi & Demo ^26^, que indicaram a relação entre a satisfação no trabalho e o comprometimento organizacional afetivo de profissionais em diferentes campos de atuação, com a diferença de que nessas últimas a relação foi examinada no sentido inverso.

Vale salientar, também, que este estudo apresentou uma contribuição importante ao demonstrar o papel de mediação desempenhado pela variável TMC, que, na grande maioria das pesquisas entre docentes, é abordada apenas como variável desfecho ^8^ ^,^ ^9^ ^,^ ^50^. Nesse sentido, os resultados aqui obtidos conduzem a refletir sobre os impactos do adoecimento mental dos docentes não apenas para o seu bem-estar, mas também para alguns resultados de interesse das organizações escolares, como o comprometimento afetivo abordado, que pode gerar desdobramentos em relação às questões de desempenho, conforme atestado por Day ^30^ e Sun ^31^, por exemplo, os quais demonstraram a relação positiva entre o comprometimento dos professores e o nível de aprendizagem dos alunos.

Ainda no que concerne aos efeitos totais das variáveis de papel na organização em relação ao comprometimento organizacional afetivo, reforçam o que já apontavam Mowday et al. ^51^, quanto ao fato de que esse último pode estar associado a características do ambiente ou a experiências de trabalho. Além disso, contribuem para fortalecer as evidências apresentadas por autores como Addae & Parboteeah ^17^, Meyer et al. ^19^ e Morrissette & Kisamore ^20^, no que tange ao conflito e à ambiguidade de papéis como antecedente do comprometimento, uma vez que foi justamente esse estressor que demonstrou maior associação com a referida variável, comparativamente à sobrecarga de papéis.

Como já salientavam Bastos et al. ^24^, a investigação dos antecedentes, correlatos e consequentes do comprometimento organizacional apresenta longa tradição nas pesquisas e este estudo contribuiu também nesse sentido, ao elucidar múltiplas relações com tal variável, apresentando elementos que auxiliam na explicação desse tipo de vínculo, o qual, nas palavras de Mowday et al. ^27^, compreende uma relação ativa na qual o indivíduo deseja contribuir em favor do bem-estar da organização.

Por fim, importa mencionar que a utilização da técnica de MEE permitiu realizar o que já assinalavam Cooper et al. ^34^ no que tange à demanda por pesquisas que explorem as inter-relações estabelecidas entre as diferentes variáveis que descrevem as reações aos estressores ocupacionais. Nesse sentido, destacam-se as potencialidades do uso da referida técnica para a identificação e a análise dos processos de mediação entre tais variáveis e a possibilidade que conferiu de acessá-las numa perspectiva de multicausalidade, que permitiu apreender melhor a complexidade das associações estabelecidas nos processos de produção de saúde e bem-estar (ou mal-estar) nas organizações de trabalho.

Deve-se atentar, todavia, para algumas limitações do estudo, entre as quais estão o emprego de amostragem não probabilística por conveniência, o qual requer cuidado no trato com os resultados, que não podem ser generalizados, ainda que o tamanho amostral obtido tenha sido relativamente elevado, considerando-se a população do estudo. Salienta-se, igualmente, o uso de abordagem transversal, que não faculta a apreensão do processo evolutivo do quadro em análise e implica uma composição amostral que envolveu apenas os docentes em exercício à época do levantamento de informações por meio dos questionários, não contemplando aqueles afastados por adoecimento.

Apesar das limitações elencadas, este estudo propiciou reunir algumas evidências sobre os efeitos danosos dos estressores de papéis para os docentes pesquisados, os quais, em termos de conflito e ambiguidade de papéis, ocorreram tanto de forma direta, em relação à satisfação laboral, aos TMC e ao comprometimento organizacional afetivo, quanto de forma indireta sobre essas últimas, com a mediação da satisfação laboral. Além disso, o efeito combinado entre a sobrecarga de papéis e o conflito trabalho-família em relação à satisfação laboral e aos TMC lança luz sobre outro aspecto da produção de mal-estar entre os sujeitos da pesquisa, convidando a refletir, a partir do cenário da educação pública nacional, sobre a relevância de que as instituições escolares se atentem para a saúde mental e o bem-estar dos docentes, favorecendo o fortalecimento de sua vinculação afetiva ao espaço de trabalho.
